# Application of Deamidated Gliadin Antibodies in the Follow-Up of Treated Celiac Disease

**DOI:** 10.1371/journal.pone.0136745

**Published:** 2015-08-31

**Authors:** Luc de Chaisemartin, Tchao Meatchi, Georgia Malamut, Fahima Fernani-Oukil, Frédérique Hosking, Dorothée Rault, Fabienne Bellery, Christophe Cellier, Marie-Agnès Dragon-Durey

**Affiliations:** 1 Immunology Department, Georges Pompidou European Hospital, Assistance Publique-Hôpitaux de Paris, Paris, France; 2 INSERM UMR 996, Paris Sud University, Châtenay-Malabry, France; 3 Pathology Department, Georges Pompidou European Hospital, Assistance Publique-Hôpitaux de Paris, Paris, France; 4 Gastroenterology Department, Georges Pompidou European Hospital, Assistance Publique-Hôpitaux de Paris, Paris, France; 5 Paris Descartes University, Paris, France; University Hospital Llandough, UNITED KINGDOM

## Abstract

**Introduction:**

The role of serological tests such as IgA anti-transglutaminase autoantibodies has become increasingly important in celiac disease (CD) diagnosis. However, the efficiency of these tests for patient follow-up is controversial. We investigated the correlation of 12 different serological tests, including recent deamidated gliadin and actin IgA tests, with villous atrophy (VA) in a retrospective cohort of treated celiac patients.

**Materials and Methods:**

Serum samples were collected from 100 treated CD patients who had intestinal biopsy in the course of their follow-up. Antibodies against transglutaminase, deamidated gliadin peptides, and native gliadin were measured, along with IgA anti-actin. The biopsy slides were all blind-reviewed and scored according to Marsh classification.

**Results:**

For all deamidated gliadin and transglutaminase tests, we found that a positive result was significantly associated with persistence of intestinal VA, with a diagnostic efficacy up to 80%. Furthermore, antibodies titers directly correlated with the degree of VA, indicating a strong link between disease activity and presence of antibodies in the serum. Interestingly, the tests with the highest association with persistent VA were those for deamidated gliadin IgG. Using a test positivity pattern analysis, we were also able to identify several groups of patients with distinct antibody profiles that showed significant differences in intestinal damage and diet compliance.

**Conclusions:**

Altogether, these results show that deamidated gliadin antibodies are strongly correlated with VA and should be considered valuable tools in CD follow-up and that multiplex serologic analysis for treated CD represents a promising tool for personalized patient management.

## Introduction

Celiac disease (CD) is an intestinal auto-immune disease, the particularity of which is to be triggered by an exogenous antigen composed of peptides from gluten in genetically susceptible individuals[1]. Clinical manifestations vary widely in type and intensity and can lead to severe complications such as osteoporosis or malignant proliferation[2]. The only treatment currently available is a life-long gluten-free diet (GFD). While the gold standard for diagnosis is the presence of a villous atrophy (VA) pattern on small bowel biopsy, the role of serological tests such as IgA anti-transglutaminase autoantibodies (IgA anti-tTG), has become increasingly important[3, 4]. Indeed, it is now possible to diagnose CD without biopsy in children having high risk of the disease and high autoantibodies levels[5].

However, in the course of adult disease follow-up, small bowel biopsies are common, either to confirm diagnosis, to assess diet efficiency or to detect refractory celiac disease. Therefore, a serological test correlating with villous atrophy in treated patients could reduce the number of endoscopic procedures needed. Despite the good performance of IgA anti-tTG antibodies in CD diagnosis, their efficiency for patient follow-up is less well documented. Under GFD, IgA anti-tTG titers decrease rather quickly in most patients, whereas it can take up to 2 years to observe normalization of the intestinal mucosa in adults, particularly in patients with high autoantibody titers[6]. Of note, some follow-up studies have shown weak association between IgA anti-tTG and villous atrophy[4, 7–9]. Recently, new specific markers of CD have been developed, in particular IgA anti-actin and antibodies against deamidated gliadin peptides. It has been suggested that IgA anti-actin titers, despite the moderate sensitivity of the test, present a close correlation with degree of VA in untreated patients[10–12]. Additionally, antibodies against deamidated gliadin peptides have in most studies shown equivalent diagnostic performance as IgA anti-transglutaminase[13–19].

We thus aimed to assess the efficiency and usefulness of these different serological tests in treated patient follow-up.

We performed 12 different serological tests on a retrospective cohort of treated celiac adult patients at a center specializing in celiac disease and correlated the results with the analysis of intestinal biopsies performed concurrently in the course of disease follow-up.

Our results show a strong association of some tests, particularly tests detecting IgG anti-deamidated gliadin, with biopsy results. Indeed, for most tests, antibodies levels correlated clearly with VA degree and GFD compliance. We then discuss the usefulness of these serological tools, alone or in combination, in celiac disease follow up.

## Materials and Methods

### Patients

A retrospective study was performed on serum samples paired with a concurrent small bowel biopsy performed in the context of celiac disease follow-up between September 2008 and March 2012. The samples were the ones collected during patients standard care procedures and no extra sample was collected for this study. After collection, all samples were routinely kept frozen (-20°C) until use. All the biopsies were performed by upper endoscopy at the Georges Pompidou European Hospital Endoscopy Unit. The initial diagnosis criteria for CD patients were the presence of villous atrophy on an intestinal biopsy sample associated with positivity of anti-transglutaminase IgA in a clinical context compatible with celiac disease. For patients with IgA deficiency, IgG anti-transglutaminase were used. The GFD compliance of treated patients was assessed during standard care procedures by a single dietician specialized in CD follow-up. The compliance data was abstracted from the medical records for this study and linked to serological and histological data. The data was then anonymized and the correspondence table destroyed. Patient records/information was anonymized and de-identified prior to analysis, therefore no consent was necessary. This study was specifically approved by the local Ethics Committee of Georges Pompidou European Hospital (AP-HP)

### Serological Tests

The characteristics of the serological tests used in this study are summarized in Table 1. We used indirect immunofluorescence, ELISA and immmunoluminescence tests, as detailed below. Indirect immunofluorescence is a microscopic test that aims to visually assess the fixation of antibodies on specific tissues or cells by means of a fluorescent secondary antibody (anti-immunoglobulin). The test can produce semi-quantitative results if end-point titration is used.

**Table 1:** Name and characteristics of serological tests.

Abbreviation	Full name	Antigen	Manufacturer
**TTg IgA EL**	Euro-tTG IgA	human recombinant tranglutaminase	Eurospital
**TTg IgA BF**	QUANTA Flash tTG IgA	human recombinant tranglutaminase	Inova
**TTg-GP IgG**	Tissue Transglutaminase IgG Assay	human recombinant tranglutaminase crosslinked with deamidated gliadin peptides	Bio-Rad
**TTg IgG**	QUANTA Flash tTG IgG	human recombinant tranglutaminase	Inova
**nGliadin IgG**	IgG anti-native gliadin	native gliadin	in-house(antigen from Sigma)
** GAF-3x IgG**	IgG anti-GAF-3x(Gliadin Analog Fusion peptide)	deamidated gliadin peptides	Euroimmun
** GAF-3x IgA**	IgA anti-GAF-3x(Gliadin Analog Fusion peptide)	deamidated gliadin peptides	Euroimmun
**DPG Screen**	QUANTA Flash DGP Screen	deamidated gliadin peptides	Inova
**DPG IgA**	QUANTA Flash DGP IgA	deamidated gliadin peptides	Inova
**DPG IgG**	QUANTA Flash DGP IgG	deamidated gliadin peptides	Inova
**Actin IgA EL**	QUANTA Lite F-Actin IgA	F-actin	Inova
**Actin IgA IF**	F-Actin (immunofluorescence)	Rat enterocytes	Eurospital

ELISA and immunoluminsescence are closely related techniques that measure the presence of specific antibodies by means of an antigen-coated plastic well, and a secondary antibody (anti-immunoglobulin) conjugated to an enzyme. The presence of the specific antibodies is measured by production of a colored (ELISA) or luminescent (immunoluminescence) substrate by the enzyme. While both techniques produce quantitative results, the luminescence technique has a greater dynamic range than classical ELISA.

### Indirect immunofluorescence

The positivity and titer of IgA anti-F-actin (named anti-actin IgA IF in this study) were evaluated semi-quantitatively by immunofluorescence with the commercial kit “F-Actin” (Eurospital, Trieste, Italy) following the manufacturer’s instructions. Briefly, rat enterocytes fixed on a glass slide were incubated 30 minutes with patient serum diluted 1:5. After washing, a FITC-conjugated anti-human IgA antibody (Eurospital, ready-to-use) was added for 30 minutes. Then, after washing and mounting, the slides were read immediately by 2 operators skilled in immunofluorescence reading. The fluorescence intensity of actin microfilaments was evaluated according to the following score: negative (0), weakly positive (1), positive (2), strongly positive (3). Discordant readings were reviewed by a third operator to reach a consensus.

### ELISA

Samples were tested by ELISA techniques for IgG anti-transglutaminase cross-linked with deamidated gliadin peptides (named tTG-GP IgG in this study, “Tissue Transglutaminase IgG Assay”, Bio-Rad, Marne-la-Coquette, France), IgA anti-transglutaminase (named anti-tTG IgA EL in this study, Euro-tTG IgA, Eurospital, Trieste, Italy), IgG anti-native gliadin (named anti-nGliadin IgG, in-house method with a gliadin antigen from Sigma Aldrich, St Quentin Falavier, France), IgG and IgA anti-GAF-3x (Bioadvance, Bussy-Saint-Martin, France) and anti-Actin IgA (named anti-actin IgA EL in this study, QUANTA Lite F-Actin IgA, INOVA Diagnostics, San Diego, CA). All tests were performed according to manufacturer’s recommendations. The procedures were realized manually for IgA anti-tTG, tTG-GP IgG, IgA anti-actin, IgG anti-gliadin or on a CARISμ instrument (Theradiag, Marne la vallée, France) for IgA and IgG GAF-3X. Results were given in arbitrary units per mL (AU/mL).

### Immunoluminescence

Serum samples were evaluated for anti-DGP IgA and IgG (QUANTA Flash DGP IgG and IgA), anti-DGP Screen (detecting both IgA and IgG simultaneously) (QUANTA Flash DGP Screen), anti-transglutaminase IgA (named anti-tTG IgA BF for disambiguation) and IgG (QUANTA Flash tTG IgA and IgG) by a chemiluminescent method on a BIO-FLASH immunoanalyzer (INOVA Diagnostics, San Diego, CA), according to manufacturer recommendations. Results were given in arbitrary units per mL (AU/mL).

### Histological evaluation

Slides from paraffin-embedded intestinal biopsy samples obtained from at least 5 different sites (1 at the bulb and 4 at distant duodenum) were all reviewed by a single pathologist (TM) skilled in intestinal pathology and scored according to Marsh-Oberhüber classification using an intraepithelial lymphocytes cut-off of 30/100[20]. Reviewing was performed blind, without any knowledge of the previous pathology reports, results of serological tests or clinical features. The patients were thereby classified into normal (Marsh 0), subnormal (Marsh I-II), partial VA (Marsh IIIa) and severe VA (Marsh IIIb-IIIc) groups.

### Statistical Analysis

Independent groups of continuous data were compared using Mann-Whitney (2 groups) or Kruskal-Wallis tests (3 groups and more). Categorical data were compared using Chi-squared test. Correlations between continuous variables were calculated by Spearman rank correlation test. Trend analyses were realized by Cuzick test for trends. A p value of less than 0.05 was considered statistically significant. Hierarchical clustering was performed using Euclidian distance and complete clustering method with Genesis 1.7 Software (Institute for Genomics and Bioinformatics, Gratz, Austria). Statistical analyses were performed using GraphPad PRISM 5.0 (GraphPad Software inc.) and MedCalc 11.1 (MedCalc Software Bvba).

## Results

### Study cohort

The study cohort consisted of 100 serum samples paired with a concurrent small bowel biopsy from treated celiac disease patients who had been referred to a single specialized outpatient center for celiac disease follow-up. The median time between initiation of diet and gastroscopy procedure was 4.5 years (range: 0.5–29). The age, sex-ratio, GFD parameters and biopsy results for all patients are summarized in Table 2. These data were compared between celiac patients with or without villous atrophy, and no significant differences between the 2 groups were observed.

**Table 2:** Population characteristics.

	Study cohort			Reference groups	
	Treated CD without VA	Treated CD with VA	Total	Non-CD	Untreated CD
***Patients (n)***	32	68	100	12	10
***Sex ratio (F/M)***	3.0 (24/8)	3.9 (54/14)	3.5 (78/22)	3.0 (9/3)	1 (5/5)
***Age median (range)***	38 (19–70)	38 (16–81)	38 (16–81)	37 (18–65)	46 (21–75)
**Diet assessment**					
***Mean time under GFD (years)***	5.2	6.9	6.9	-	-
***GFD for >2 years***	69% (22)	63% (43)	65% (65)	-	-
***GFD for < 2 years***	31% (10)	37% (25)	35% (35)	-	-
**Biopsy result**					
***Normal***	18	-	18	10	0
***Subnormal***	14	-	14	0	0
***Partial Atrophy***	-	39	39	2	3
***Severe atrophy***	-	29	29	0	7

Two control groups were also constituted, consisting of untreated celiac patients (n = 10) and patients in whom CD had been excluded by intestinal biopsy (n = 12), to be used only as reference populations in antibody positivity pattern analysis (these control groups were used only in the part of the Results section regarding antibody profile analysis). Demographical data of these groups did not differ significantly from the main patient group. All untreated CD patients presented a VA pattern, and all non-celiac patients had a normal mucosa except for two with mild VA. Two patients presented total IgA deficiency and were therefore excluded from analysis of IgA detecting tests.

### Serological tests discriminate treated CD patients with villous atrophy

**Fig 1:**
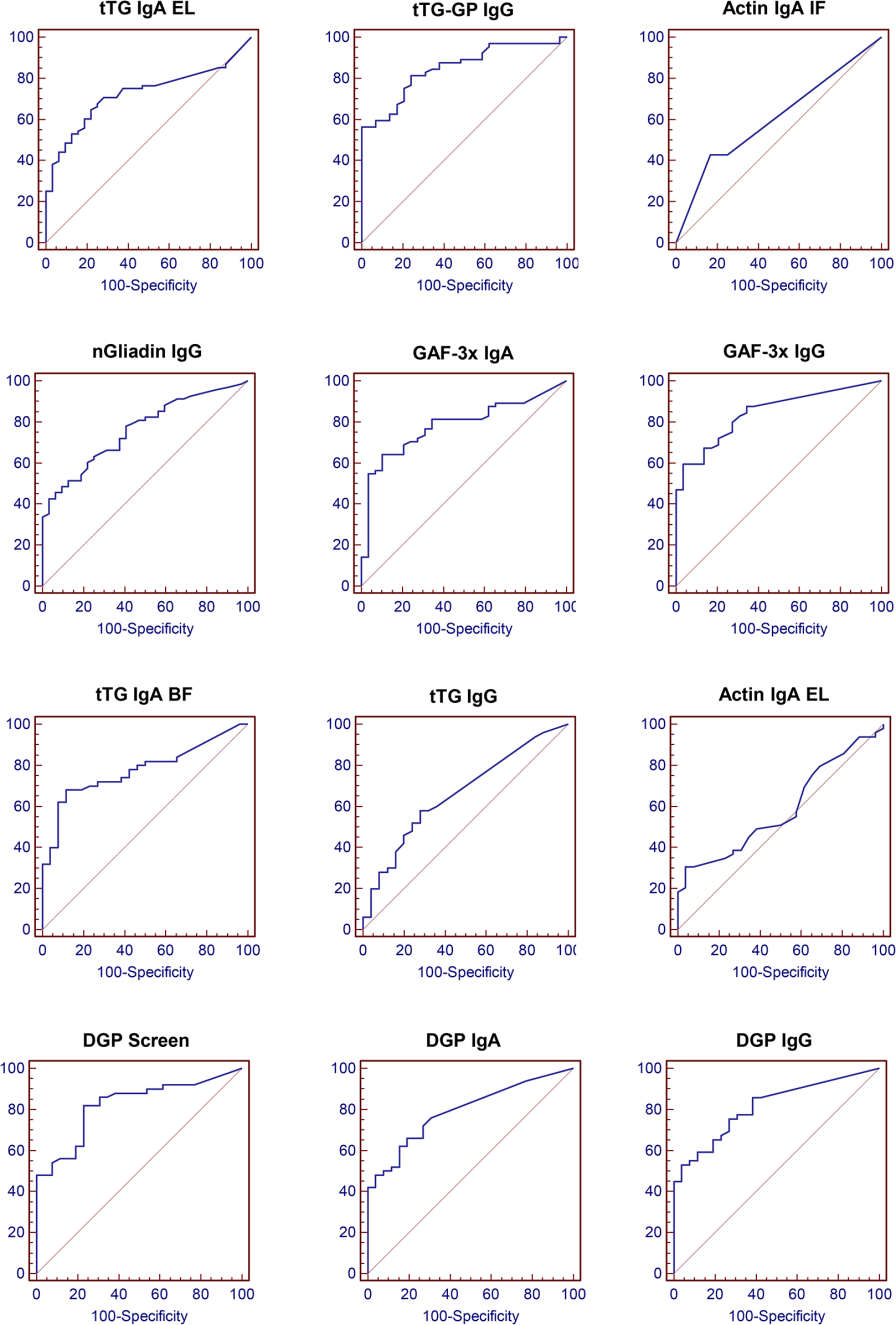
Adaptation of serological tests to treated CD patients. ROC curve analysis was performed on treated celiac patients by plotting sensitivity against 100-specificity for all test values. The presence of VA was used as the positivity criteria. The area under curve (AUC) and its statistical significance were calculated for each curve and are summarized in Table 3.

Currently available kits for serological diagnosis of celiac disease are mainly designed for screening previously undiagnosed patients. Consequently, manufacturer-provided cut-off values are calculated using untreated celiac patients as positive control population. To assess whether the analytical performance of tested antibodies would allow efficient discrimination of treated CD patients with or without persistent VA, we first verified if these cut-off values could be used for a treated patient population. A ROC (Receiver-Operating Characteristic) curve analysis was thus performed on our treated CD patient group for each test using presence of intestinal VA as the positivity criteria (Fig 1). Test performance, as measured by the AUC (Area Under Curve) of the ROC curves, ranged from 0.587 to 0.848 (Table 3).

**Table 3:** Analytical performance of serological tests.

			*Optimal*				*95% Specificity*	
								
	AUC	p	Sensitivity	Specificity	Efficacy	Cut-off	Sensitivity	Cut-off
**tTG-GP IgG**	0.848	1.14x10^-17^	81.2	75.9	79.5	3.9	56.3	11.8
**GAF-3x IgG**	0.844	6.20x10^-18^	59.4	96.6	71.0	21.0	59.0	21.0
**DGP Screen**	0.822	9.07x10^-11^	82.0	76.9	80.2	4.1	48.0	14.8
**DGP IgG**	0.813	1.30x10^-10^	53.1	96.2	68.0	17.9	53.6	17.6
**DGP IgA**	0.792	1.05x10^-8^	66.0	80.8	71.0	7.1	48.0	13.6
** GAF-3x IgA**	0.782	2.08x10^-8^	64.1	89.7	72.1	19.0	54.7	36.0
**tTG IgA BF**	0.777	3.68x10^-7^	68.0	88.5	75.0	9.5	40.0	50.0
**nGliadin IgG**	0.763	8.78x10^-8^	42.6	96.9	60.0	85.0	42.6	85.0
**tTG IgA EL**	0.725	1.73x10^-5^	64.7	78.1	69.0	4.0	38.2	25.0
**tTG IgG**	0.661	0.0112	58.0	72.0	62.7	4.0	20.0	15.8
**IgA Actin IF**	0.607	0.1674	42.9	83.3	55.8	0.0	0.0	0.0
**IgA Actin EL**	0.587	0.1959	30.6	96.2	51.6	22.0	30.6	22.0

Recalculation of optimal cut-off values showed that differences could be observed between the manufacturers’ recommended cut-off and the calculated optimal cut-off for our population (S1 Fig). We thus used the calculated optimal cut-off obtained for the treated CD patient population for all subsequent analysis.

Statistical analysis of the ROC curves showed significant ability to discriminate VA-positive from VA-negative patients for all studied tests except for the two anti-actin IgA tests (Table 3). As a result, the latter two were not studied further. Tests using deamidated gliadin peptides as an antigen (GAF-3x, DGP and tTG-GP antibodies) were compared to the other tests (tTG and nGliadin antibodies) and presented significantly higher AUC (mean 0.817±0.03 versus 0.732±0.05, p = 0.009)(Table 3). Since sensitivity was an important feature of the tests in our setting, we also compared them at a fixed 95% specificity, which allowed comparison for sensitivity. Deamidated gliadin peptide tests were found to have significantly higher sensitivities (mean 53% versus 35.2%, p = 0.002). Interestingly, among deamidated gliadin tests, those with the highest AUC all detected IgG isotype (tTG-GP IgG, GAF-3x IgG, DGP Screen and DGP IgG).

These results show that serological tests for celiac disease are able to discriminate patients with persistent VA in a treated patient population. Of note, antibodies against deamidated gliadin peptides, particularly of IgG isotype, had the highest AUC in a ROC-curve analysis.

### Correlation between antibodies positivity and intensity of intestinal damage

**Fig 2:**
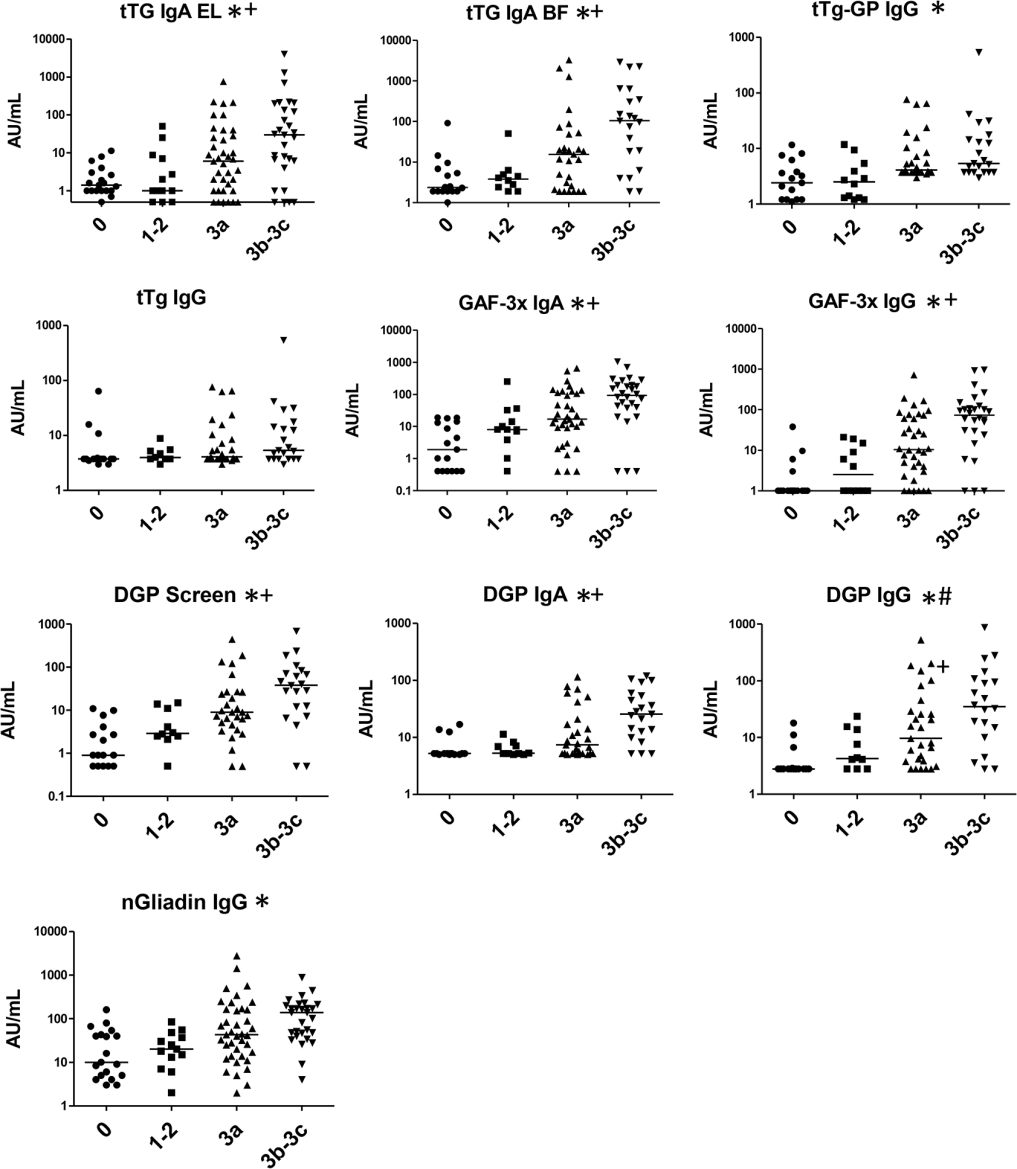
Association of serological tests with degree of intestinal damage. Antibody titers for each test were plotted for groups of patients with different intestinal damage as defined by the Marsh score. The horizontal line represents the median titer for each test. (*): the titers rise significantly with degree of intestinal damage (Kruskal-Wallis and Cuzick trend tests). (+): the titers are significantly higher in severe versus partial atrophy groups (Mann-Whitney test). (#): the titers are significantly higher in subnormal versus normal biopsy groups (Mann-Whitney test).

Having determined that serological tests were able to discriminate patients with VA in a treated CD patient population, we assessed if there was a correlation between test results and intestinal damage. For this analysis, intensity of intestinal damage was measured by Marsh-Oberhüber classification as described in the Methods section (Fig 2).

Antibody titers were found to differ significantly among the various intestinal damage groups (p = 0.0014 to p<0.0001, Kruskal-Wallis test) for all tests except tTG IgG test. Moreover, a strong positive correlation between the titers and VA intensity could be observed (p<0.0001, Cuzick trend test, S1 Table).

This correlation between antibody titers and biopsy results was then further tested to assess if it was strong enough to specifically discriminate patients with partial versus severe atrophy, and patients with normal from subnormal biopsy. When compared in this way, tTG IgA, GAF-3x IgA and IgG, DGP IgA and DGP Screen had significantly higher titers in severe (Marsh IIIb-c) versus partial atrophy (Marsh IIIa) (tTG IgA EL: 48±21 vs 257±143, p = 0.04; tTG IgA BF: 251±137 vs 484±189, p = 0.02; GAF-3X IgA: 78±24 vs 170±43, p = 0.005; GAF-3x IgG: 55±21 vs 148±46, p = 0.005; DGP Screen: 41±17 vs 84±33, p = 0.03; DGP IgA: 21±5 vs 39±8, p = 0.009). However, no significant difference could be observed between patients with normal versus subnormal biopsies (Marsh 0 versus Marsh I-II) except for DGP IgG test (4±1 vs 8±2, p = 0.03).

Altogether, these results show that antibodies titers, with the exception of tTG IgG, are strongly linked with the severity of intestinal damage.

### Antibody profile defines groups of patients with distinct intestinal damage

The relevance of combined antibody detection in a single individual was then investigated by analyzing the test positivity profile for individual patients and correlating this profile with clinical parameters.

**Fig 3:**
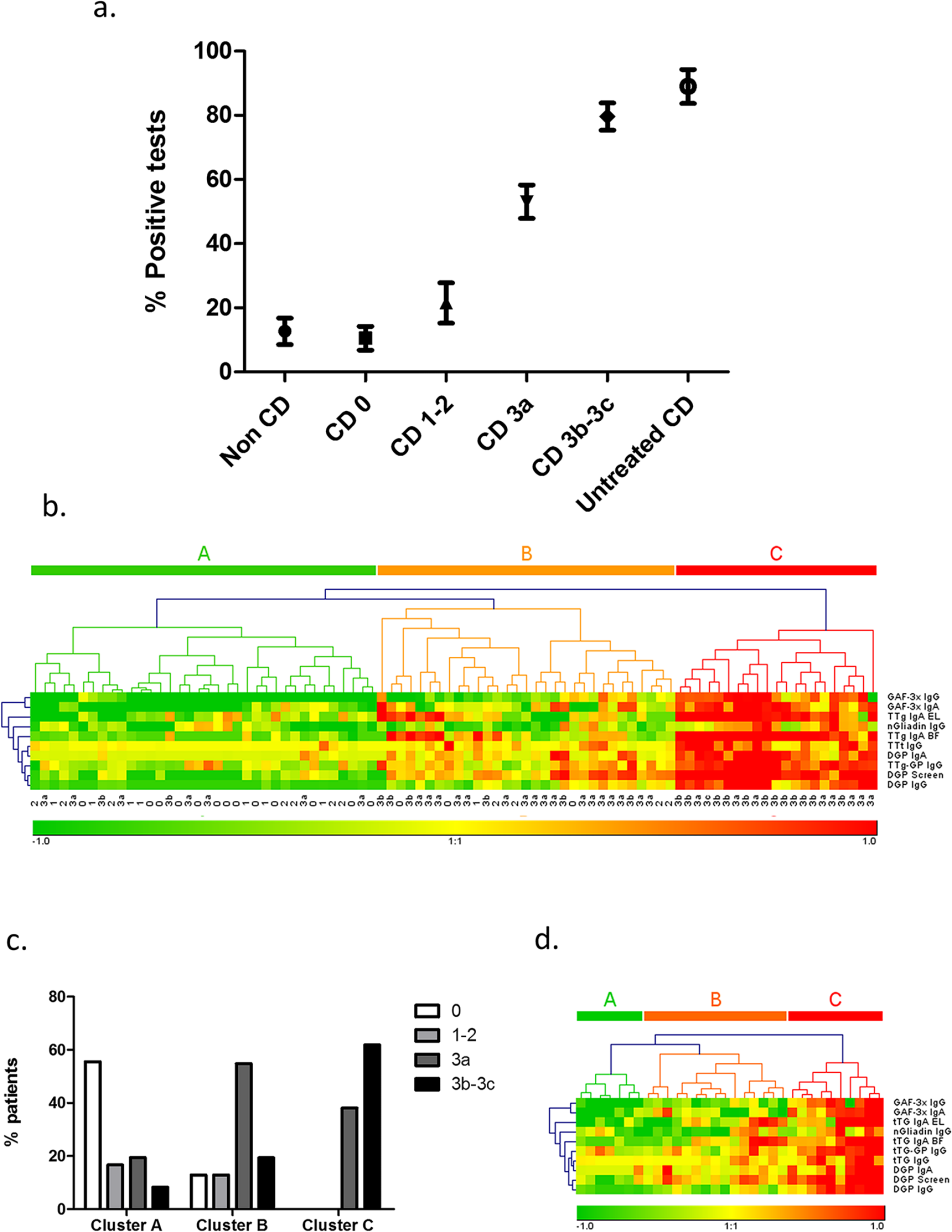
Analysis of antibody positivity profile. (a): Percentage of positive tests (Mean ±SEM) for each patient group. (b): Unsupervised hierarchical clustering of serological data. Each square represents the result of one test (lines) for one patient (columns), while its color represents the positivity level of the test. The test titers were normalized by calculating the ratio between titer and cut-off. Antibody titers close to cut-off values are represented by yellow squares, titers below cut-off range from yellow (cut-off) to green (very low titers) and titers above cut-off range from yellow (cut-off) to red (very high titers). (c): Proportion of histological scores in the patient clusters. (d): Unsupervised hierarchical clustering of serological data restricted to patients with partial atrophy (Marsh 3a).

First, the percentage of positive tests was assessed for each patient and plotted against patient’s clinical status (Fig 3A). We observed that the percentage of positive tests progressively rose from 11% for CD patients with normal biopsies to 80% for patients with Marsh 3b-3c biopsies. These proportions were compared to those obtained for our 2 reference groups of non-celiac patients and untreated celiac patients (as described in the beginning of the Results section). When compared this way, treated CD patients with normal or subnormal biopsies (Marsh 0 and Marsh 1–2) were not significantly different from non-coeliac controls (p = 0.1) and treated CD patients with severe atrophy were not different from untreated CD patients. (p = 0.2).

However, the percentage of positive test results was significantly different between CD patients with partial atrophy versus subnormal biopsies (p = 0.003) and between CD patients with severe versus partial atrophy (p = 0.0006).

Altogether, these data show that the number of positive tests for a single individual is strongly and positively correlated with the intensity of intestinal damage (Spearman coefficient r_s_ = 1.0, p = 0.017).

We then hypothesized that not only the number of positive antibody but also their type would help separate the patients into groups of distinct antibody profiles. Serological data was thus submitted to unsupervised hierarchical clustering in order to sort patients into groups according to similarity of antibody profile (Fig 3B). Using this technique, 3 major clusters of patients could be observed (A, B, C from left to right). The patients in cluster A were negative for the majority of tests, while those in cluster C had high titers for most antibodies and those in cluster B had a mixed profile. The clinical relevance of these self-sorted patient clusters was then tested by comparing patients’ histological features. In agreement with the results shown in Fig 3A, normal biopsy patients were clearly predominant in cluster A (p<0.00001), partial atrophy patients were predominant in cluster B (p = 0.02), while cluster C contained significantly more patients with severe atrophy (p = 0.004) and no patient without VA (Fig 3C). Subnormal biopsies were present only in clusters A and B, but in too small a number to be analyzed.

Since partial atrophy patients were predominant and found in all 3 clusters, we checked if the analysis of the antibody profile could also define groups of patients in a population with the same level of intestinal damage. The same hierarchical clustering technique was applied again to patients with Marsh 3a biopsies. Despite a loss of precision due to a smaller number of patients, 3 patient clusters were easily distinguishable with distinct antibody positivity patterns (Fig 3D), suggesting the existence of patient subgroups amongst patients with a common Marsh 3a biopsy.

Altogether, these analyses suggest that antibody profile analysis could help sorting patients into clinically relevant groups and thereby provide new and useful data for CD patient monitoring.

### Antibody profile correlates with gluten intake

**Fig 4:**
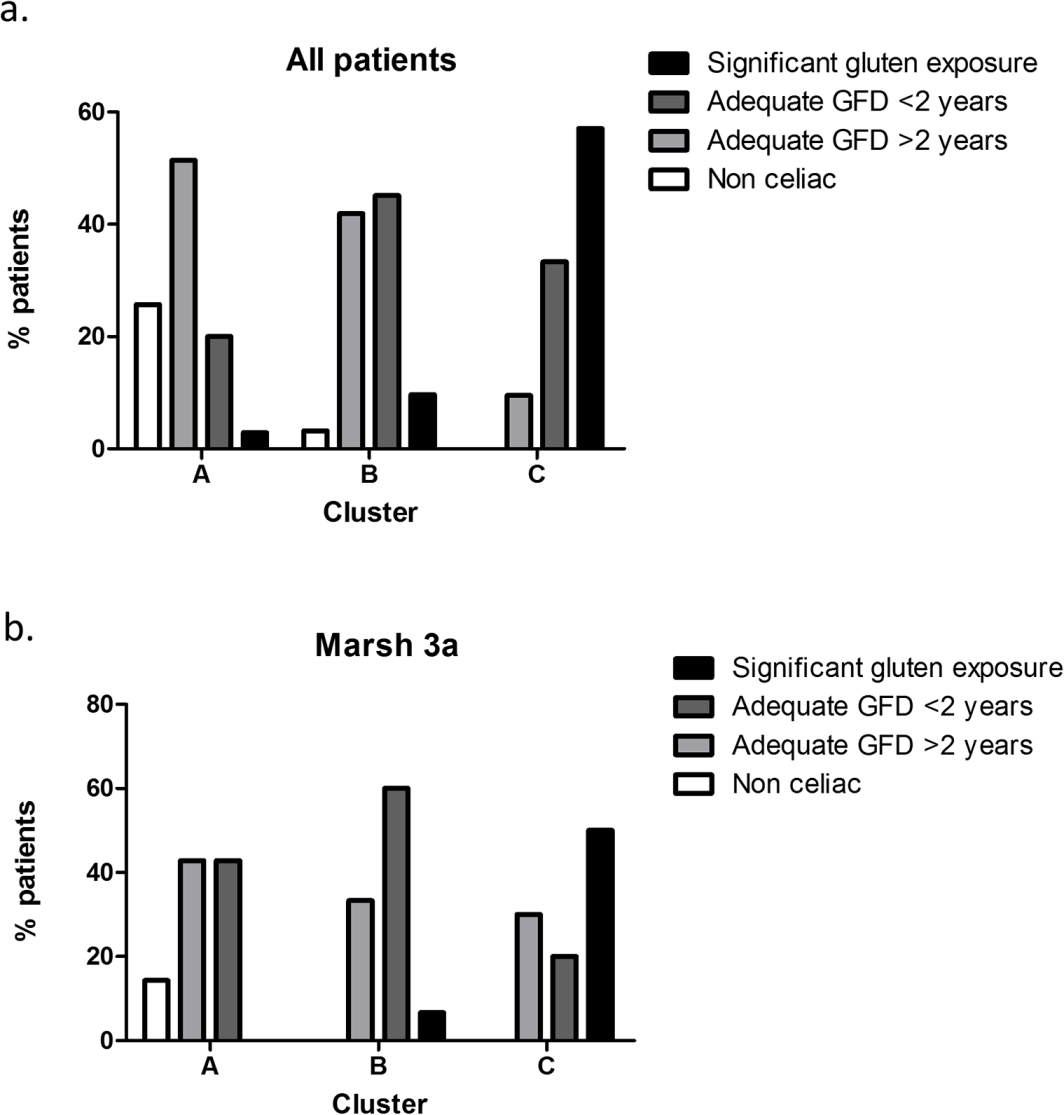
Correlation of patient clusters with gluten intake. Proportion of patients with significant gluten intake, GFD for less than 2 years, GFD for more than 2 years in antibody-defined patient clusters A, B and C, calculated in Fig 3(A): All clustered patients, (b): Partial atrophy (Marsh 3a) patients.

It has previously been shown that villous atrophy does not correlate well with clinical symptoms but is associated with gluten intake in CD patients[21, 22]. For this reason, we analyzed gluten exposure for each patient cluster, based on diet data collected by a specialized dietician (Fig 4A). Since intestinal recovery under GFD can take up to 2 years in adults[6], the CD patients were divided into 3 groups according to their gluten intake: a group of patients with significant gluten intake (including all patients declaring any measurable amount of gluten intake), a second group with patients under strict GFD for less than 2 years, and a third group with patients under GFD for more than 2 years. The non-celiac reference population was also analyzed for comparison. We found that patients with adequate GFD for more than 2 years as well as non-celiac controls were predominantly in cluster A (p = 0.004 for both) while patients with significant gluten intake were predominantly in cluster C (p = 0.0005, Chi square test). The patients with more recent GFD were mostly in cluster B but could also be found in clusters A and C, reflecting the progressive process of mucosa healing under treatment.

The same analysis was done for partial atrophy patients with similar results (Fig 4B). In this group, patients with significant gluten intake were mostly in cluster C while patients on GFD for less than 2 years were predominant in cluster B. Patients with long-term GFD could be found in all clusters with similar proportions (30% to 42%). However, the number of patients in some categories did not allow statistical analysis.

Together, these data show that patient groups defined by antibody profile have significant differences in gluten intake, confirming further the link between antibody profile and intestinal damage suggested by our previous analysis.

### Comparison of paired test sensitivity

Our patient classification by antibody profile analysis was based on 10 simultaneous serological tests. In a clinical setting, performing such a large number of tests would simply not be practical for CD patient follow-up. Consequently, we focused our efforts on identifying a smaller, more practical set of tests that would still provide effective serological screening of treated CD patients for VA persistence. The tests were grouped into a total of 45 pairs, and the sensitivity of each pair for persistence of intestinal VA was calculated. The five best combinations achieved a sensitivity of 88% (Table 4). Interestingly, all these combinations included at least one deamidated gliadin test, and all but one included both IgG and IgA measurement. We propose that such test combinations could be useful in screening treated CD patients for persistent VA who would most benefit from a follow-up biopsy, and that further prospective studies on the matter should reevaluate the need for invasive procedures in this setting.

**Table 4:** Analytical performance of paired serological tests for association with VA persistence on intestinal biopsy in treated patients.

Test combination		Sensitivity	Specificity
DPG Screen	tTG IgA BF	87.8	73.1
DPG Screen	tTG IgA EL	87.8	69.2
tTG-GP IgG	DPG Screen	88.0	65.4
tTG-GP IgG	tTG IgA BF	87.8	65.4
tTG-GP IgG	tTG IgG	88.0	56.0

## Discussion

In this study, 12 different serological tests, including 6 deamidated gliadin tests were directly correlated with residual intestinal damage assessed by a follow-up intestinal biopsy in a cohort of treated adult CD patients. Our results clearly show that a majority of the tests have significant association with persistence of VA, with an efficacy up to 80%. Furthermore, test results significantly correlated with intensity of intestinal damage, indicating a strong link between disease activity and presence of antibodies in the serum. The only exceptions were IgA-actin, which displayed poor sensitivity (30% for ELISA and 43% for IFI) consistent with previous reports in untreated celiac patients[23], and tTG IgG, for which titers correlated poorly with intestinal damage.

These efficacy figures may seem somewhat low as compared to the better performance of serology in untreated CD patients; however, treated patients are a much more complex population with great individual variation in diet compliance and treatment response. Accordingly, we believe that treated CD patient monitoring is an important aspect of clinical practice that needs to be addressed in serological studies.

We found that the tests with the highest association with persistent VA in treated CD patients were deamidated gliadin-based tests, particularly of IgG isotype. This is in line with some reports on untreated CD patients, suggesting superiority of deamidated gliadin for CD diagnosis[18, 24, 25] or even GFD monitoring[17, 26, 27]. In the only study to our knowledge directly comparing biopsy result and deamidated gliadin test in treated patients, Volta et al. found in a group of 53 treated adult celiac patients that DGP IgA, DGP IgG and tTG IgA could detect patients with persistent VA after 1 year of GFD (n = 14 villous atrophy; sensitivities 67%, 60% and 67%, specificity 79%, 89% and 76%, respectively)[14]. While we found similar performance for the same tests on a larger cohort with a higher percentage of patients with VA (68% versus 26%), our work shows that tTG-GP IgG, GAF-3x IgG and DGP Screen tests which in our study obtained sensitivity up to 81% for tTG-GP IgG and specificity up to 97% for GAF-3x IgG at optimal cut-off, perform better.

The importance of the IgG isotype may seem surprising since IgA are usually considered the best markers for celiac disease. Indeed, IgA tTG clearly outperform IgG tTG for CD diagnosis; however, for deamidated gliadin antibodies, a slight advantage of the IgG isotype for CD diagnosis in untreated patients has been reported, though not much emphasized, in several studies[15, 28–30].

Taken together, our results suggest that gliadin-derived tests, particularly of IgG isotype, are associated with VA persistence with a strong specificity and should be considered valuable tools in celiac disease follow-up despite their low sensitivity.

While some previous studies on CD antibodies analyzed up to 6–7 different tests[16, 31], these tests were considered individually instead of providing an overall positivity profile analysis for single individuals. Furthermore, it has been shown that neither transglutaminase nor DGP are able to cross-inhibit binding of CD patient antibodies, suggesting that they represent non-cross-reacting antibodies populations[19, 32]. Consistently, we found that treated celiac patients display various positivity profiles, indicating that even if the antigens used in the tests are related, the antibodies populations detected do not completely overlap. Interestingly, we observed that the number of positive antibodies rises alongside with the intensity of intestinal damage, which could be interpreted as an intensification of the immune response associated with a broadening of the recognized epitopes repertoire, or epitope spreading, as has been shown in type 1 diabetes and rheumatoid arthritis[33, 34].

To analyze further these different serological profiles, we used hierarchical clustering, a classical approach in gene expression analysis, which allows sorting a population in an unsupervised manner in groups of patients with similar expression profiles. Here, using the same method, we observed that our treated patient population could easily be divided into groups with distinct antibody positivity profiles. These groups were clinically relevant since each group had distinct VA levels and GFD status. Furthermore, we suggest that this kind of analysis could be even more accurate than Marsh grading as far as CD follow-up is concerned. Indeed, most treated patients display partial atrophy (Marsh 3a) on their intestinal biopsy, with various degrees of GFD compliance, and consequently various degrees of actual intestinal damage. Here, our analysis of antibody positivity profile was able to define distinct groups of patients with significant differences in GFD compliance, even among those with the same level of intestinal damage. This suggests that antibody profile analysis could benefit treated CD patients by identifying more accurately those with greater intestinal damage and allowing them to be referred for diet reassessment, thus limiting the risk of subsequent severe complications. Altogether, we suggest that multiplex serologic analysis in CD represents a new and interesting tool with potential for improving personalized patient care.

The concurrent measurement of 10 antibodies is currently costly and complicated in practice. Moreover, the sensitivity of each single test was not sufficient to warrant use in clinical practice on its own. We thus propose a set of 2 tests that could be helpful in serological screening of treated patients for persistence of VA. Several combinations seemed to perform adequately, all containing at least one anti-deamidated gliadin test. Interestingly, the best combinations reached a higher sensitivity than any single test. Moreover, most combinations contained both IgG and IgA tests, suggesting that in some treated patients the antibodies best associated with VA could be IgG and in others, IgA.

The retrospective setting of this study led to some limitations. First, the time between diet initiation and follow-up gastroscopy differed between patients, as some came for a relapse after several years of diet while others came for systematic follow-up around 1–2 years after diet initiation. Second, assessment of gluten intake was not done using a standardized questionnaire and thus did not allow us to study groups with different levels of gluten exposure.

To our knowledge, however, the present treated CD patient cohort with follow-up biopsy results and deamidated gliadin antibody measurements is the largest of its type published to date. With a greater number of patients, one could measure more precisely analytic performances of tests pairs on patient subgroups with distinct clinical parameters, like gluten intake. In the context of personalized medicine development, performance assessment of tests in specific clinical situations is of great importance, and we believe this question should be specifically addressed on a prospective setting.

In summary, this study shows that several serum antibodies, in particular those against deamidated gliadin, are closely associated with biopsy results in CD follow-up. We suggest that cut-off values used for untreated celiac patients are not necessarily appropriate for patient follow-up and should be adjusted to the population being tested. Furthermore, we show that the use of several tests simultaneously could help to detect treated CD patients with persistent VA and more active disease.

We believe that this preliminary work shows great potential for serology to significantly reduce the need for endoscopic procedure in treated CD patients and also provide additional and relevant information for diet assessment and patient follow-up.

## Supporting Information

S1 STARD Checklist STARD Checklist. **STARD checklist for reporting of studies of diagnostic accuracy**. Required checklist for diagnostic study quality assessment.
(DOCX)

S1 Fig Serological Tests cut-off values. This graph compares for each test the cut-off value provided by the manufacturer and the optimal cut-off value calculated with the ROC curve analysis on treated CD patient population (Fig 1).
(TIF)

S1 Table Statistical analyses of antibodies. This table provides the p-values for the statistical analyses used for evaluating serological tests in Fig 2. Numbers in bold were considered significant.
(PPT)
