# Supplementary figures and images for: Application of Deamidated Gliadin Antibodies in the Follow-Up of Treated Celiac Disease
Source: PLoS One. 2015 Aug 31;10(8):e0136745. doi: 10.1371/journal.pone.0136745 (PMC4554732; doi:10.1371/journal.pone.0136745)

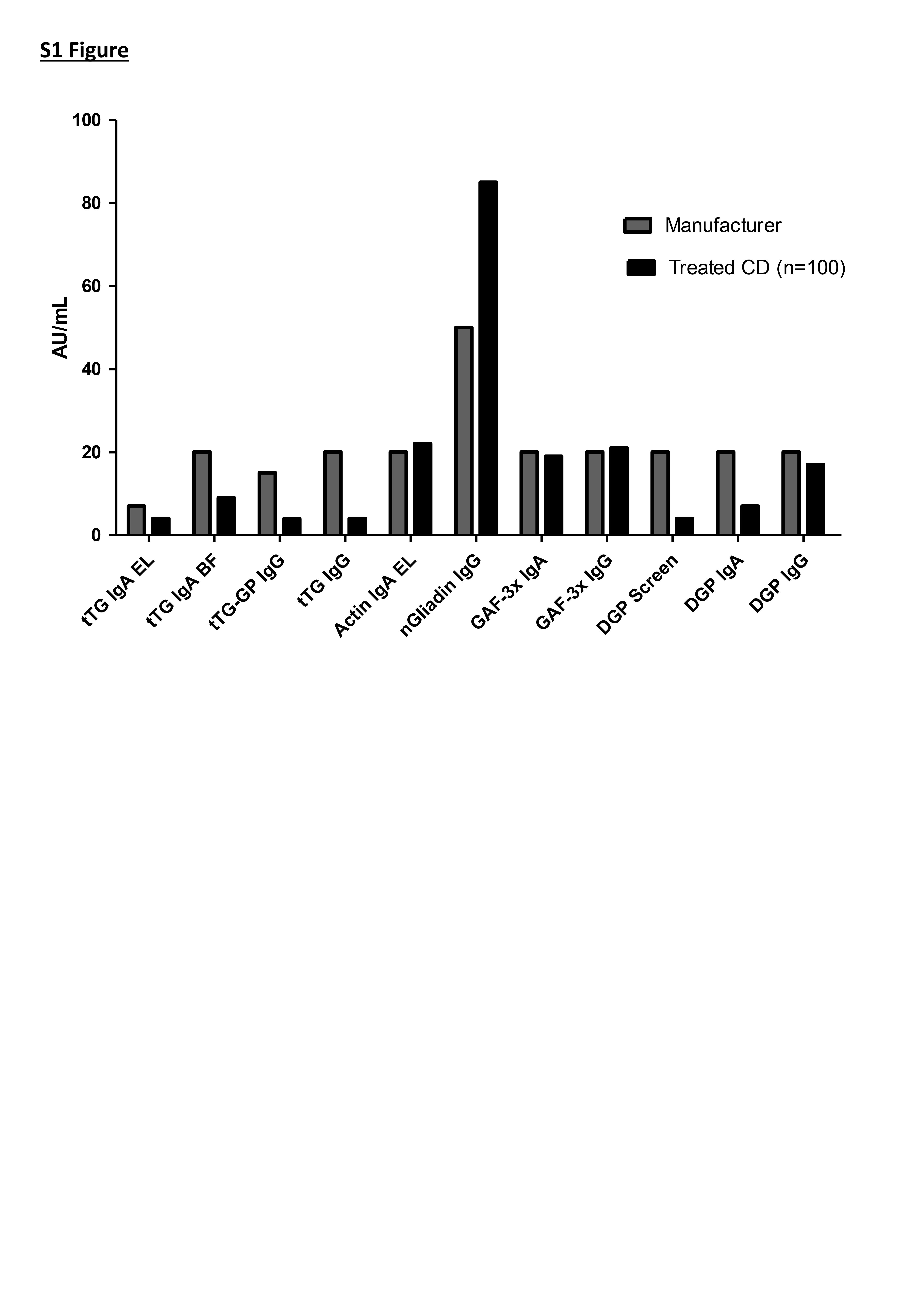

Supplement: S1 Fig — This graph compares for each test the cut-off value provided by the manufacturer and the optimal cut-off value calculated with the ROC curve analysis on treated CD patient population (Fig 1). (TIF) [file pone.0136745.s002.tif]
