# Supplementary material for: Application of Deamidated Gliadin Antibodies in the Follow-Up of Treated Celiac Disease
Source: PLoS One. 2015 Aug 31;10(8):e0136745. doi: 10.1371/journal.pone.0136745 (PMC4554732; doi:10.1371/journal.pone.0136745)
Supplement: S1 Table — This table provides the p-values for the statistical analyses used for evaluating serological tests in Fig 2. Numbers in bold were considered significant. (PPT) [file pone.0136745.s003.ppt]

## Slide 1
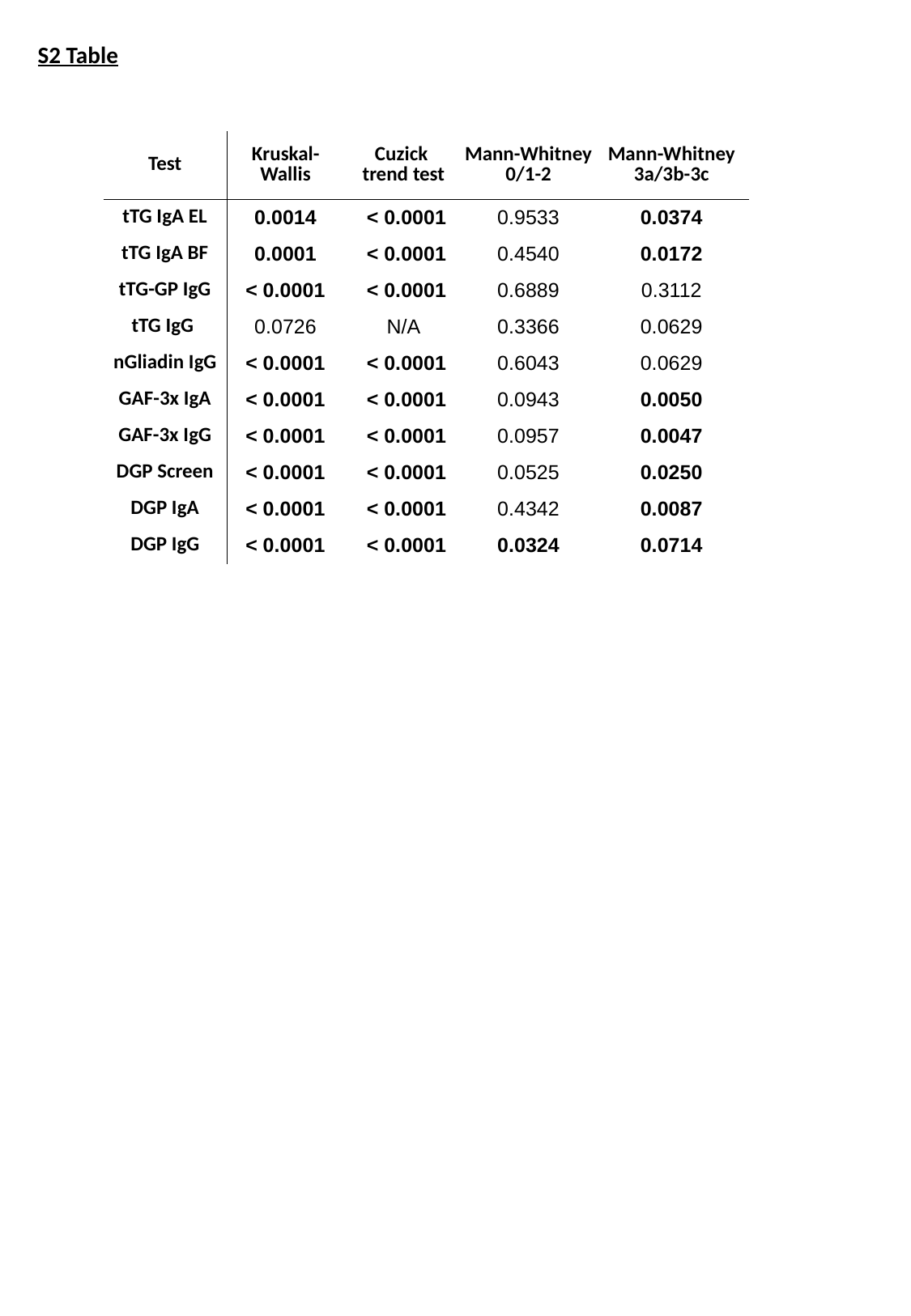

S2 Table
| Test | Kruskal-Wallis | Cuzick trend test | Mann-Whitney 0/1-2 | Mann-Whitney 3a/3b-3c |
| --- | --- | --- | --- | --- |
| tTG IgA EL | 0.0014 | < 0.0001 | 0.9533 | 0.0374 |
| tTG IgA BF | 0.0001 | < 0.0001 | 0.4540 | 0.0172 |
| tTG-GP IgG | < 0.0001 | < 0.0001 | 0.6889 | 0.3112 |
| tTG IgG | 0.0726 | N/A | 0.3366 | 0.0629 |
| nGliadin IgG | < 0.0001 | < 0.0001 | 0.6043 | 0.0629 |
| GAF-3x IgA | < 0.0001 | < 0.0001 | 0.0943 | 0.0050 |
| GAF-3x IgG | < 0.0001 | < 0.0001 | 0.0957 | 0.0047 |
| DGP Screen | < 0.0001 | < 0.0001 | 0.0525 | 0.0250 |
| DGP IgA | < 0.0001 | < 0.0001 | 0.4342 | 0.0087 |
| DGP IgG | < 0.0001 | < 0.0001 | 0.0324 | 0.0714 |
